# Diseases of the Digestive Organs

**Published:** 1905-04-08

**Authors:** 


(*To be continued.*)

---

### DISEASES OF THE DIGESTIVE ORGANS.

**The Stomach Reflex.**—Albert Abrams[1] shows that on percussing smartly with a pleximeter over the area of Traube, namely, over the cardiac end of the stomach, the whole stomach area will become dull, and can therefore be distinguished from the surrounding organs. This reflex lasts from 30 to 90 seconds, and the intensity of the dulness forms also some measure of the contractile or motor power of the stomach. The cause of this dulness depends on the tension produced in the gastric walls which interferes with the vibration of the gas contained within, thus changing the normally tympanitic note into a higher one. Percussion of the epigastrium after the reflex is started tends to abolish it, but a series of smart blows on Traube's area will revive it.

**Perforation of an Ulcer,** according to F. and G. Gross,[2] usually occurs in persons with a long previous history of stomach trouble, but in a few there have been no previous symptoms. Occasionally perforation of an ulcer arises from a strain or effort. There is sudden severe pain in the epigastrium, and often between the shoulders, and tenderness over the stomach. Vomiting is said by some to be frequent when the perforation is near the pylorus, and rare when near the cardia. The abdominal wall is rigid and generally retracted, and there is marked dulness in the flanks. It is curious that the pulse is usually good in the first 24 hours, the temperature is most uncertain, but frequently there is evidence of severe shock, after which signs of general or localised peritonitis come on. As soon as the shock permits laparotomy should be performed. Moynihan[3] remarks that the frequency with which cancer of the stomach is preceded by a history of ulcer is variously estimated. He himself thinks that such a history only occurs in 10 per cent. of the cases, but some hold it is as high as 60 per cent. and even more. Whatever estimate be correct, it is clear that cancer occurs in just those regions in which ulcer is found—that is, on the posterior surface and on the lesser curvature. Symptoms of an ulcer may have existed for many years previously, or may have appeared for the first time shortly before the cancer shows itself. In rare cases the ulcer is latent, and only found on operation. Cabot and Badger[4] hold that, while perforating ulcer of the stomach demands immediate surgical treatment, hæmorrhage can nearly always be controlled by medical means. Cases of chronic dyspepsia which tend to develop into hour-glass stomach should be operated upon before they show the complete change, and cases of chronic dilatation which do not yield to treatment, if not due to general ptosis, may also need operation. So, too, in frequently recurring or intractable hæmorrhage, or intractable dyspepsia really due to ulcer. A curious abdominal condition which may be mistaken for appendicitis or localised abscess is seen in the gastro-intestinal crises of Erythema exudativum. Pond[5] reminds us that these crises may cause attacks of colic, vomiting, rapid pulse, rigidity, and perhaps a tumour. In making a diagnosis notice should be taken of hyperæsthetic areas of œdema, bleeding from the gums, joint symptoms, urinary symptoms, and family history. Operation should be refused unless there is a rapidly increasing leucocytosis. R. C. Kemp[6] thinks that there is a tendency to rely on an examination of the gastric contents rather than on the motor functions and position of the stomach, which are really more important. Dilatation and gastroptosis are, in fact, results of motor failure. For testing the size and position of the stomach he claims that "diaphany" is more reliable than other methods. He also employs a whistle at the end of a stomach tube worked by a rubber bulb. The sounds are heard by the stethoscope, and the stomach area can be thus marked out.

**Dyspepsia.**—W. Langdon Brown[7] shows the practical value of recent discoveries. Thus the dependance of the secretion of gastric juice on nerve influences started in the palate, and its stimulation by meat extract, and to some extent by water, milk, or gelatine, shows the value of soups made of meat extracts at the beginning of a meal, and, on the other hand, the harm they do in hyperchlorhydria. Alcohol stimulates appetite, leads to increased secretion of juice, increased peristaltic movements, and increased absorption. Indeed water and aqueous solutions are hardly absorbed from the stomach at all, but are sent on through the pylorus. An excess of alcohol delays digestion, and strong solutions act as irritants causing a mucous catarrh. Starch excites no flow of juice, and the proteid of bread requires far more pepsin for its digestion than that of meat. The value of starchy food in dyspepsia is probably over estimated. The nervous factor in secretion explains too the value of "bitters" (though they have no direct action on the glands) and the wasting of patients fed through a gastric fistula. The administration of alkalies after meals except in hyperchlorydria is harmful. Though it relieves the discomfort caused by organic acids, it neutralises the HCl., and with it the pepsin. Given before meals, they inhibit secretion, and thus, perhaps, give power by enforcing rest, as digitalis does by prolonging diastole. Moreover, they dissolve mucus in catarrhal states. The real strength of HCl. in the stomach seems much greater than was believed, and is probably ·5 or ·6 per cent. in the dog. It appears to be frequently absent in cardiac and pulmonary diseases and fevers. Even the secretion of pancreatic juice fails if HCl. is not present. Hence the value of full doses of the acid in many states where it is deficient.

**Bacteria.**—E. Palier[8] made a series of experiments on the flora of the stomach and finds that in cancer there are present (1) the vibrio geniculatus, (2) many staphylococci, and that fungi are absent. In simple hypochlorhydria, the vibrio may be present, and any other organism. In hyperchlorhydria yeast and mycelia and a small bacillus not stained by Gram, but the special combination found in cancer is peculiar to it and not present in other states.

**Phlegmonous Gastritis.**—Hopkins and Weir[9] give a report of one of these rare cases in a man, aged 38, who had suffered for two months from cystitis and from some gastric pain. While convalescent in the hospital rigors and epigastric pain came on, followed by symptoms of peritonitis. Death occurred in 12 days. Under the mucous membrane of the stomach was a large quantity of pus towards the pyloric end, and two abscesses which passed through to the subserous coat, probably causing the peritonitis. The writers remark upon the difficulty of diagnosis unless a rupture of the abscess takes place into the stomach. Some 81 cases have been collected, only 17 of which were in this country. The cause of the present case is obscure since there was no focus of suppuration and no dietetic error to account for it.

**Pancreatic Cancer and Glycosuria.**—Pearce[10] has made a study of 30 cases of cancer of the pancreas, in three of which glycosuria was present, but finds that it is impossible to say from the literature what proportion of cases of cancer show glycosuria also. However, he concludes that diabetes is sometimes due to cancer involving the islands of Langerhans. The cause of intermittent glycosuria is uncertain, the absence of glycosuria in cases of cancer depends on the survival of sufficient islands to carry on the metabolism of sugar. Surviving islands become hypertrophied in an effort to do the work. The anatomical independence of the islands is shown by their persistence in the midst of the cancerous growth which destroys the acini. There is some evidence that the islands show a compensatory hypertrophy in disease of the liver. Ohlmacher[11] mentions Warthin's instance of malignant disease of the liver where the islands in the pancreas were from three to five times their normal size. He has also examined 40 cases where the liver was more or less involved by various disease processes, and found that there was great enlargement of the islands in practically all of them, and this not of an inflammatory nature. This hypertrophy has for its object the compensation of the failure of the liver in governing the carbohydrate changes. One theory is that the glycolytic ferment of the islands oxidises any excess of sugar, and that they hypertrophy with increased work during disease of the liver. Another is that they normally restrain the action of the liver in converting the glycogen it has gained, and when the liver fails to retain its glycogen additional work is thrown on the pancreas. This compensatory growth of the islands explains the fact that some diabetics, after careful dieting, acquire again the power of digesting carbohydrates without glycosuria.

[1] Med. Rec., Sept. 3. [2] Birm. Med. Rev., Nov. [3] Practit., Sept. [4] Med. Rec., Sept. 17. [5] Ibid. [6] Lancet, Nov. 19. [7] Practit., Oct. [8] Med. Rec., Nov. 19. [9] Brit. Med. Jour., Nov. 19. [10] Amer. Jour. Med. Sci., Sept. [11] Ibid., Aug.

